# Measurement properties of the EQ-5D-Y administered through a smartphone app in children with asthma: a longitudinal questionnaire study

**DOI:** 10.1186/s12955-022-01955-5

**Published:** 2022-03-28

**Authors:** Karina Mayoral, Olatz Garin, Catalina Lizano-Barrantes, Angels Pont, Araceli M. Caballero-Rabasco, Manuel Praena-Crespo, Laura Valdesoiro-Navarrete, María Teresa Guerra, José Antonio Castillo, Inés de Mir, Eva Tato, Jordi Alonso, Vicky Serra-Sutton, Yolanda Pardo, Montse Ferrer, ARCA Group

**Affiliations:** 1 Health Service Research Group, IMIM (Hospital del Mar Medical Research Institute), Barcelona Biomedical Research Park, office 144. Doctor Aiguader, 88, 08003 Barcelona, Spain; 2 Department of Paediatrics, Obstetrics and Gynaecology and Preventive Medicine, Universitat Autònoma de Barcelona, Barcelona, Spain; 3 CIBER en Epidemiología y Salud Pública, CIBERESP, Madrid, Spain; 4 Department of Experimental and Health Sciences, Universitat Pompeu Fabra, Barcelona, Spain; 5 Department of Pharmaceutical Care and Clinical Pharmacy, Faculty of Pharmacy, Universidad de Costa Rica, San José, Costa Rica; 6 Paediatric Pulmonology and Allergy Unit, Paediatric Department, Hospital Del Mar, Barcelona, Spain; 7 Centro de Salud La Candelaria. Servicio Andaluz de Salud, Sevilla, Spain; 8 Grupo de Vías Respiratorias de La Asociación Española de Pediatras de Atención Primaria (AEPAP), Madrid, Spain; 9 Hospital Universitari Parc Taulí, Sabadell, Spain; 10 Centro de Salud de Jerez Sur, Jerez de la Frontera, Spain; 11 Hospital Miguel Servet, Zaragoza, Spain; 12 Hospital Vall d’Hebron, Barcelona, Spain; 13 Hospital Universitario Araba, Vitoria-Gasteiz, Spain; 14 Agency for Health Quality and Assessment of Catalonia, Barcelona, Spain; 15 Department of Psychiatry and Legal Medicine, Universitat Autònoma de Barcelona, Barcelona, Spain

**Keywords:** Health-Related Quality of Life, Asthma, EQ-5D-Y, Validity, Reliability, Responsiveness, Smartphone app

## Abstract

**Background:**

Asthma impacts children’s physical, emotional, and psychosocial Health-Related Quality of Life (HRQL). The EQ-5D-Y is a generic econometric instrument developed to measure HRQL in children.

**Objective:**

Evaluation of feasibility, validity, reliability, and responsiveness of EQ-5D-Y descriptive system and utility index to allow the assessment of HRQL in children with asthma, aged 8–11 years (self-response version) or under 8 years old (proxy-response version).

**Methods:**

We used data from baseline to 10 months of follow-up of an observational, prospective study of children with persistent asthma recruited by pediatricians in Spain (2018–2020). HRQL instruments were administered through a smartphone application: ARCA app. The EQ-5D-Y is composed of a 5-dimension descriptive system, a utility index ranging from 1 to − 0.5392, and a general health visual analogue scale (EQ-VAS). The Pediatric Asthma Impact Scale (PROMIS-PAIS) includes 8 items, providing a raw score. Construct validity hypotheses were stated a priori, and evaluated following two approaches, multitrait–multimethod matrix and known groups’ comparisons. Reliability and responsiveness subsamples were defined by stability or change in EQ-VAS and the Asthma Control Questionnaire (ACQ), to estimate the intraclass correlation coefficient (ICC) and the magnitude of change over time.

**Results:**

The EQ-5D-Y was completed at baseline for 119 children (81 self-responded and 38 through proxy response), with a mean age of 9.1 (1.7) years. Mean (SD) of the EQ-5D-Y utility index was 0.93 (0.11), with ceiling and floor effects of 60.3% and 0%, respectively. Multitrait–multimethod matrix confirmed the associations previously hypothesized for the EQ-5D-Y utility index [moderate with PROMIS-PAIS (0.38) and weak with ACQ (0.28)], and for the EQ-5D-Y dimension “problems doing usual activities” [moderate with the ACQ item (0.35) and weak with the PROMIS-PAIS item (0.17)]. Statistically significant differences were found in the EQ-5D-Y between groups defined by asthma control, reliever inhalers use, and second-hand smoke exposure, with mostly moderate effect sizes (0.45–0.75). The ICC of the EQ-5D-Y utility index in the stable subsamples was high (0.81 and 0.79); and responsiveness subsamples presented a moderate to large magnitude of change (0.68 and 0.78), though without statistical significance.

**Conclusions:**

These results support the use of the EQ-5D-Y as a feasible, valid, and reliable instrument for evaluating HRQL in children with persistent asthma. Further studies are needed on the responsiveness of the EQ-5D-Y in this population.

**Supplementary Information:**

The online version contains supplementary material available at 10.1186/s12955-022-01955-5.

## Introduction

Asthma is a chronic condition that affects more than 300 million people worldwide [1], and it is the most common chronic disease during childhood, affecting around 14% of children globally [2]. Patient-reported outcome measures (PROMS), such as symptom control or Health-Related Quality of Life (HRQL), have been shown to be useful for clinical management, in combination with clinical measures, providing relevant information to understand the impact of the disease on patients’ functional status and well-being [3–5]. Given its heterogeneous nature and symptoms burden, asthma has physical, emotional, and psychosocial impact on children’s lives, as has been shown through diverse generic HRQL instruments [6–10]. The most affected dimensions of asthma-specific HRQL instruments are peer relationships, feeling of dependence on medication, shortness of breath, and activity limitations [11–13]. There is consistent evidence that HRQL and asthma control are independent predictors of future exacerbation [14–17]. Furthermore, a systematic review [18] found that asthma severity was significantly related to the child’s HRQL in most of the studies. International guidelines [19–22] have emphasized that treatment goals should focus on improving the day-to-day symptoms of the patient, preventing exacerbations, and improving patients’ HRQL.

HRQL instruments are generic or specific according to their target population, and they can in turn be classified as psychometric profiles or econometric indexes according to their measurement model [23]. Psychometric instruments generate scores on different dimensions in order to describe them (profiles). Econometric measures provide a single global score (index) which incorporates societal preferences for health states (utilities) that can be used to calculate quality-adjusted life years for use in economic evaluations [24]. The EQ-5D has probably been the most widely used econometric instrument in adults, and the EuroQol Group developed in 2010 the EQ-5D-Y to enable young individuals from 8 years onwards to self-report their health [25–28]. An EQ-5D-Y proxy version was also developed [29] for children under 8 years old.

There are few other econometric questionnaires for children, such as the Health Utility Index (HUI) and the Child Health Utility 9D (CHU-9D). The HUI [30] has a self-administered version and a proxy version (children 5–12 years old), but its administration burden is substantially greater than for the EQ-5D-Y. The interviewer-administered EQ-5D-Y showed high feasibility and agreement with the CHU-9D among 6–7 years old children [31], and several studies supported the acceptability [32], feasibility [32, 33], reliability [33], validity [33, 34], and responsiveness [35] of the EQ-5D-Y self-administered version in children and adolescents from the general population aged 8–18 years. Furthermore, the psychometric properties of the EQ-5D-Y have already been tested in several pathologies, such as chronic kidney disease [36], cystic fibrosis [37], juvenile idiopathic arthritis [38], type 1 diabetes mellitus [39], idiopathic scoliosis [40], and chronic or acute conditions [41]. As far as we know, there is only one study centered on the psychometric properties of the EQ-5D-Y in patients with asthma [42], supporting its feasibility and its convergent validity with the Pediatric Asthma Quality of Life Questionnaire. Other studies in heterogeneous samples that included children and adolescents with asthma [43, 44] also supported the EQ-5D-Y’s feasibility, reliability, and construct validity.

There is extensive evidence on the reliability [45], construct validity [45, 46] and responsiveness [47] of EQ-5D in adults, both for the descriptive system and the utility index. However, all previous studies on EQ-5D-Y in children evaluated only the dimensions of the descriptive system, or an equally weighted summary score. None of them evaluated the EQ-5D-Y utility index, because the value set for children has just been published [48]: first for the Slovenian [49] and then for the Spanish [50] population.

To the best of our knowledge, this is the first study evaluating the psychometric properties of the EQ-5D-Y utility index, and also the first one assessing the psychometric properties of the EQ-5D-Y proxy version for children with asthma < 8 years of age. We have found only two studies supporting the reliability and construct validity of the EQ-5D-Y proxy version, both performed in the general population [29, 51].

Our aim was to evaluate the feasibility, validity, reliability, and responsiveness of the EQ-5D-Y descriptive system and utility index to allow the assessment of Health-Related Quality of Life in children with asthma aged 8–11 years old (self-response version) or under 8 years old (proxy-response version).

## Methods

### Participants and study design

The Asthma Research in Children and Adolescents (ARCA) is a longitudinal prospective multicenter observational study (NCT04480242), designed to provide evidence about the evolution of young patients with persistent asthma through a regular follow-up.

Patients were recruited in 3 outpatient pediatric pulmonology hospital units and 8 primary care pediatric centers in Spain, from January 2018 to July 2020. Families were informed about the project and asked to participate if their children fulfilled the following inclusion criteria: aged 6–14, with clinical diagnosis of asthma, undergoing treatment with inhaled corticosteroids (alone or combined with long-acting beta-agonists) for more than 6 months in the previous year, and with access to a smartphone (their own, or their parents’). Exclusion criteria were: chronic obstructive pulmonary disease, cystic fibrosis, pulmonary fibrosis, bronchiectasis, active tuberculosis, or/and immunodeficiency associated with alpha 1 antitrypsin deficiency, ciliary diseases.

### Study variables

The ARCA study collects information through different sources: medical records, computer-assisted telephone interviews performed by trained interviewers, and the ARCA smartphone application [52]. The EQ-5D-Y and the Patient-Reported Outcomes Measurement Information System-Pediatric Asthma Impact Scale (PROMIS-PAIS) were administered through the ARCA app, while all the variables to define known groups for validity assessment were collected through telephone interviews, and clinical characteristics came from medical records.

The ARCA app development has been described elsewhere [52]. Briefly, HRQL questionnaires are administered every 6 months: the EQ-5D-Y at baseline and month 6 of follow-up, and the PROMIS-PAIS at months 4 and 10. The ARCA app is available in 3 age versions (6–7, 8–11, and ≥ 12 years old) following the EQ-5D age cut-off points. The version for the younger age group was designed to be answered by parents or guardians (proxy response), and the other two versions for children’s and adolescents’ self-response. For the evaluation of the EQ-5D-Y, 12–14 years old adolescents were excluded because the EQ-5D-5L is administered in this age version.

The EQ-5D-Y was developed to measure HRQL in children [25]. It includes a descriptive system [26] of 5 dimensions and a visual analogue scale (EQ-VAS) of general health. The dimensions measure “mobility”, “looking after myself”, “doing usual activities”, “having pain or discomfort” and “feeling worried, sad or unhappy” with 3-level Likert response scales (no problems, moderate problems, and serious problems). The EQ-VAS ranges from 0, worst health possible, to 100, best health possible. The time frame for both the dimensions and the EQ-VAS is “today”. The EQ-5D-Y proxy version 1, which asked proxies to rate the child’s HRQL in their own opinion, has the same characteristics as the self-reported version. Evidence on the Spanish EQ-5D-Y’s validity, feasibility, and reliability has been reported [33].

From the three digital versions of the EQ-5D-Y, laptop/desktop, tablets, and PDA/smartphone, we administered the latter through a smartphone app [52] with its original generic content, without including any expression for asthma-specific attribution. The preference value set to generate the EQ-5D-Y utility index for Spain [48] was obtained from adults thinking as a hypothetical 10-year-old child, as recommended in the international protocol. A single preference-based index was calculated ranging from 1 (the best health state) to negative values (health states valued by society as worse than death), where 0 is equal to death.

The Patient-Reported Outcomes Measurement Information System (PROMIS) developed a disease-specific item bank to measure the HRQL of children with asthma [53]: the Pediatric Asthma Impact Scale (PROMIS-PAIS). The short form 8a version of the PROMIS-PAIS (v2.0) contains the item set that provides the maximum test information with the least items [52]. It has demonstrated a higher precision [53] than other asthma-specific instruments [54, 55], while presenting a lower administration burden. Each item of the PROMIS-PAIS is attributed to asthma with the expression “because of my asthma”, except for the last one which states “My asthma bothered me”. The items [56] ask about the past seven days in a 5-level Likert response scale (1–5) with the options: never, almost never, sometimes, often, and almost always. It is available for self-response in ages 8–17, and for proxy response in children starting at age 5. The total raw score is calculated by adding the values of the response to each question, the lowest possible score is 8 and the highest is 40. Missing items were imputed by a simple allocation method from the mean of those items that were available in each dimension of the questionnaire [57].

The information collected through telephone interviews included, among other, the Asthma Control Questionnaire (ACQ), exacerbations occurring in the previous 6 months, treatments for asthma, and secondhand smoke exposure. Two versions of telephone interviews were developed, one designed to be answered by parents or guardians of children under 8 years old (proxy response) and the other for self-response (participants aged 8 and older).

The Asthma Control Questionnaire ACQ-symptoms only [58] assesses the frequency of 5 asthma symptoms (night-time waking, symptoms on waking, activity limitation, shortness of breath, and wheeze) during the previous week on a 7-level Likert scale from 0 (no impairment) to 6 (maximum impairment). The overall score, calculated as the mean of item responses, ranges from 0 to 6. Cut-off points of 1.5 and 0.75 are established to define not well- and well-controlled asthma, respectively [59]. Results generated by this short version have shown to be very similar to those of the complete ACQ, as were its measurement properties (reliability, responsiveness, internal consistency, construct validity, and interpretability) [58]. The ACQ has been validated [60] using self-administration in children 11 years and older and interviewer-administration in 6- to 10-year-olds.

Asthmatic exacerbations during the last 6 months were assessed through three questions constructed applying the definitions by the American Thoracic Society and the European Respiratory Society [61]: *Did you visit or phone your family doctor or outpatient emergency department because your asthma got worse? Did you call an ambulance or go to the hospital because of your asthma? Did you take steroids tablets or syrup (such as Prednisolone or Deltacortril) for at least 2 days because of your asthma?* If the participant answers “yes” to at least one of the three questions, an asthma exacerbation is confirmed.

To measure the frequency of Short-Acting Beta-Agonists (SABA) inhaler use during the previous 4 weeks, the following question was asked to patients with SABA therapy prescription: *How often have you usually taken your “reliever medication” (brand name) in the past 4 weeks?* (Every day; almost every day; once or twice every week; less than once a week; or I don’t know).

Secondhand smoke exposure was measured through a single question taken from the High School Risk Factors Survey [62]: *How many people out of those who stay in your house regularly smoke indoors?* (No one smokes indoors; 1 person; 2; 3; 4; 5 people and more than 5 people).

### Ethics considerations

The study was approved by the ethics committee of participant centers in accordance with national and international guidelines (code of ethics, Helsinki Declaration) as well as legislation on data confidentiality (Spanish Organic Law 3/2018 of December 5 on the Protection of Personal Data and the Guarantee of Digital Rights). The collection and transfer of data was carried out according to strict security and data encryption. Written informed consent was required from the parents or legally authorized representatives of all participants, and additionally oral consent was obtained from children.

### Analytical strategy

Considering the ARCA sample of 119 patients, a statistical power of 80% (using a two-side test with a type I error of 5%) was calculated to detect moderate differences (0.5 SD) in the EQ-5D-Y utility index between two equally distributed known groups, or to detect moderate to large differences (0.65 SD) between two known groups unequally distributed into 85% and 15% of the sample [63].

Characteristics of the sample were described by calculating percentages, or means and standard deviations, according to the type of variable. To evaluate the feasibility of the EQ-5D-Y, we calculated the completion rate, the distribution of the response options, and the proportion of missing values. Distribution of the EQ-5D-Y utility index, EQ-VAS, and the PROMIS-PAIS raw score were examined by calculating the observed range, the floor and ceiling effects (proportion of participants with the worst and best possible score, respectively), and statistics of central tendency and dispersion.

Construct validity of the EQ-5D-Y was assessed by applying two different approaches: multitrait-multimethod matrix, and comparison of known groups. The multitrait-multimethod matrix between the EQ-5D-Y, the PROMIS-PAIS, and the ACQ was constructed with Spearman correlations due to the scores’ distribution. Besides their scores, the dimension or item on activities was also included in the matrix, since it was covered by all three questionnaires. The strength of the correlations was defined [64] as weak (≤ 0.30), moderate (0.31–0.60), or strong (0.61–0.90).

The relationships between instruments can be categorized as convergent (different instruments measuring a similar concept) or discriminant (different instruments measuring different traits or constructs). For convergent validity, we hypothesized a moderate correlation between the PROMIS-PAIS raw score and the EQ-5D-Y utility index, since both instruments intend to measure HRQL though from different perspectives (generic and asthma-specific). Also, we expect moderate correlations between the EQ-5D-Y dimension “problems doing usual activities”, and the ACQ item “how limited were you in your activities because of your asthma?”. On the other hand, for divergent validity, we expected a weak correlation between the EQ-5D-Y utility index and the ACQ, since they differ on the construct being measured (HRQL and disease control). A weak correlation was also expected between the EQ-5D-Y dimension “problems doing usual activities” and the PROMIS-PAIS item “it was hard for me to play sports or exercise because of my asthma”, due to differences in the type of activities considered.

Known groups were defined according to the ACQ (well-controlled, intermediate, and not well-controlled asthma) [59], asthmatic exacerbation during the last 6 months (yes or no), frequency of SABA inhaler use during the previous 4 weeks (less than once vs once or more per week), and secondhand smoke exposure (exposed or not). The hypotheses raised a priori, based on available evidence, were that patients with worse control of asthma [65], asthmatic exacerbations [66], higher frequency of SABA use [65], and second-hand smoke exposure [67] present worse HRQL. In particular, we expected the EQ-5D-Y to present worse discriminant capacity than the disease-specific PROMIS-PAIS because of its generic nature. To assess differences among groups we used Chi-square test for proportions of participants with problems, and U-Mann Witney or Kruskall-Wallis nonparametric test for the HRQL scores. The magnitude of the differences between groups was assessed by the Cohen effect size (difference of mean/pooled SD) [68]. General guidelines define an effect size of 0.2 as small, 0.5 as moderate, and 0.8 as large [69].

To assess reliability and responsiveness, patients were divided into three subsamples defined according to stability, worsening or improvement between the two administrations of the EQ-VAS and the ACQ. On one hand, patients who changed in EQ-VAS ± 0.3 SD or less (small magnitude) were included in the stable subsample, and those with a change larger than 0.3 SD (moderate or large magnitude) in the negative and positive direction were considered for the worsening and improvement subsamples respectively. The cut-off point of 0.3 SD [69] was selected following the established interpretation of the magnitude of change. On the other hand, according to the ACQ, patients that remained in the same asthma control category were included in the stable subsample, while those which moved to a worse or better category were included in the worsening or improvement subsample.

Since our hypothesis was that a stable EQ-VAS or ACQ indicates health stability over time, test–retest reproducibility of the EQ-5D-Y and PROMIS-PAIS was assessed in the stable subsamples by measuring the agreement between the two administrations with the Intra-class Correlation Coefficient (ICC). Regarding responsiveness, our hypothesis was that the EQ-5D-Y is able to detect change over time, though with a lower sensitivity than the asthma-specific instrument PROMIS-PAIS. Responsiveness was evaluated in the worsening and improvement subsamples by testing differences between the two administrations in the EQ-5D-Y or PROMIS-PAIS with the Wilcoxon paired test. The magnitude of change was measured by the effect size coefficient (mean of change/SD of change) for the worsening and improvement subsamples analyzed together. IBM SPSS Statistics software, version 22, was used to analyze the data.

## Results

**Fig. 1:**
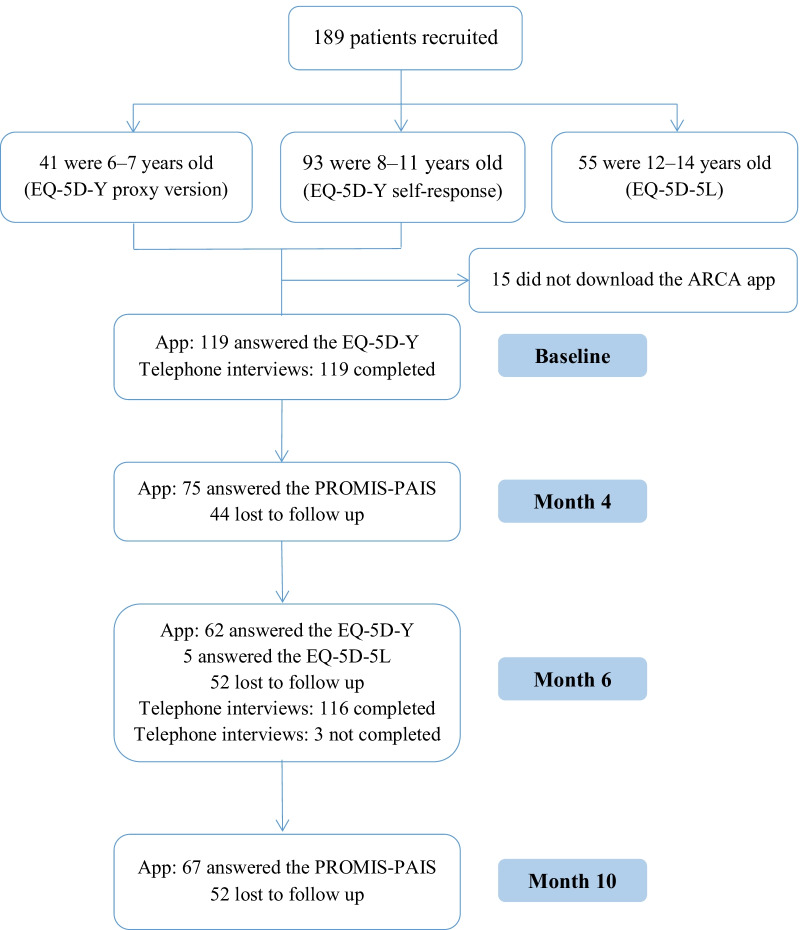
Flow chart of the study from recruitment to month 10

Of the 189 participants recruited (see Fig. 1), 55 patients aged 12–14 years were excluded because they were administered the EQ-5D-5L, 15 did not download the ARCA app, and 119 in total were included in the study: 81 were 8–11 years old children who completed the self-response version, and 38 were 6–7 years old children whose parents or guardians completed the proxy response version of the EQ-5D-Y. Table 1 shows the characteristics of the sample at baseline. Around 60% of the participants were boys and had well-controlled asthma. Differences between age groups were only found for asthmatic exacerbation, which was more frequent among 6 to 7-year-olds.

**Table 1:** Demographic and clinical characteristics of participants at baseline

	All (n = 119)	EQ-5D-Y Self response (n = 81)	EQ-5D-Y Proxy response (n = 38)	*P* value
*Age*, mean (SD)	9.1 (1.7)	10.1 (1.1)	7.0 (0.6)	< .001
6–7	38 (31.9%)	0 (0.0%)	38 (100.0%)	< .001
8–11	81 (68.1%)	81 (100.0%)	0 (0.0%)	
*Sex*				
Girls	48 (40.3%)	29 (35.8%)	19 (50.0%)	.14
Boys	71 (59.7%)	52 (64.2%)	19 (50.0%)	
*Asthma control*				.99
ACQ^a^, mean (SD)	0.81 (0.93)	0.81 (0.88)	0.81 (1.06)	
Well controlled (< 0.75)	68 (58.6%)	43 (54.4%)	25 (67.6%)	.37
Intermediate (0.75–1.5)	23 (19.8%)	18 (22.8%)	5 (13.5%)	
Not well controlled (> 1.5)	25 (21.6%)	18 (22.8%)	7 (18.9%)	
*Asthmatic exacerbations (last 6 months)*				
Yes	53 (44.5%)	27 (33.3%)	26 (68.4%)	< .001
No	66 (55.5%)	54 (66.7%)	12 (31.6%)	
*Number of prescribed SABA*^b^				
0	9 (7.8%)	6 (7.6%)	3 (8.1%)	.72
1	103 (88.8%)	71 (89.9%)	32 (86.5%)	
2	4 (3.4%)	2 (2.5%)	2 (5.4%)	
*Frequency of SABA inhaler use (previous 4 weeks)*				
No use	9 (7.8%)	6 (7.6%)	3 (8.1%)	.99
Less than once per week	62 (53.4%)	43 (54.4%)	19 (51.4%)	
Once or twice per week	29 (25.0%)	19 (24.1%)	10 (27.0%)	
Almost every day/Every day	16 (13.8%)	11 (13.9%)	5 (13.5%)	
*Secondhand smoke exposure*				
Not exposed	81 (83.5%)	55 (84.6%)	26 (81.3%)	.92
Exposed	16 (16.5%)	10 (15.4%)	6 (18.7%)	
Missing	22	16	*6*	

^a^ACQ: Asthma Control Questionnaire

^b^SABA: Short-Acting β-Agonists

Very few patients reported problems in the EQ-5D-Y dimensions (Table 2), especially for “mobility” (5.1%) and “looking after myself” (2.6%). The dimension showing the highest percentage of participants with problems was “doing usual activities” (28.8%) where there was 1 participant reporting “a lot of” problems. Very few missing values were observed in some EQ-5D-Y dimension (“mobility”, “looking after myself”, and “doing usual activities”).

**Table 2:** Distribution of EQ-5D-Y dimensions in the entire sample at baseline

EQ-5D-Y dimensions	No problems	Some problems	A lot of problems	Missing values
	n (%)	n (%)	n (%)	n (%)
Mobility	111 (94.9%)	6 (5.1%)	0 (0.0%)	2 (1.7%)
Looking after myself	114 (97.4%)	3 (2.6%)	0 (0.0%)	2 (1.7%)
Doing usual activities	84 (71.2%)	33 (28%)	1 (0.8%)	1 (0.8%)
Having pain or discomfort	98 (82.4%)	21 (17.6%)	0 (0.0%)	0 (0.0%)
Feeling worried, sad or unhappy	110 (92.4%)	9 (7.6%)	0 (0.0%)	0 (0.0%)

At month 4 after recruitment (when the PROMIS-PAIS was administered through the app), 44 participants were lost to follow-up, and 75 answered the PROMIS-PAIS. Table 3 shows the results of the participants who answered the PROMIS-PAIS (self-response n = 59; proxy response n = 16). Patients reported more frequently “my asthma bothered me” and “it was hard to play sports or exercise because of asthma”. The PROMIS-PAIS was completed entirely by the responders, with no missing values for any of the items.

**Table 3:** Distribution of the Pediatric Asthma Impact Scale (PROMIS-PAIS) items’ responses at month 4 after recruitment

PROMIS-PAIS^a^ items	Never	Almost never	Sometimes	Often	Almost always
	n (%)	n (%)	n (%)	n (%)	n (%)
I felt scared that I might have trouble breathing because of my asthma	60 (80.0%)	8 (10.7%)	6 (8.0%)	1 (1.3%)	0 (0.0%)
My chest felt tight because of my asthma	53 (70.7%)	13 (17.3%)	7 (9.3%)	1 (1.3%)	1 (1.3%)
I felt wheezy because of my asthma	52 (69.3%)	8 (10.7%)	14 (18.7%)	1 (1.3%)	0 (0.0%)
I had trouble breathing because of my asthma	52 (69.3%)	12 (16.0%)	9 (12.0%)	2 (2.7%)	0 (0.0%)
I had trouble sleeping at night because of my asthma	56 (74.7%)	9 (12.0%)	10 (13.3%)	0 (0.0%)	0 (0.0%)
It was hard for me to play sports or exercise because of my asthma	48 (64.0%)	12 (16.0%)	9 (12.0%)	5 (6.7%)	1 (1.3%)
It was hard to take a deep breath because of my asthma	52 (69.3%)	13 (17.3%)	8 (10.7%)	2 (2.7%)	0 (0.0%)
My asthma bothered me	46 (61.3%)	14 (18.7%)	13 (17.3%)	2 (2.7%)	0 (0.0%)

^a^PROMIS-PAIS: Patient-Reported Outcomes Measurement Information System-Pediatric Asthma Impact Scale

As shown in Table 4, the mean (SD) of the EQ-5D-Y utility index in the total sample was 0.93 (SD = 0.11). This high mean is explained by the accumulation of patients in 1, the highest score (best HRQL). This ceiling effect of 60.3% is caused by the high number of participants reporting “no problems” in all dimensions. Despite this accumulation in the highest score, the observed range (1–0.5095) indicates a high variance. The EQ-VAS mean (SD) was 84.3 (17.1) among the whole sample. There were three patients with a missing value in the EQ-5D-Y utility index, while the EQ-VAS was completed by all responders. The PROMIS-PAIS raw score mean (SD) was 11.9 (4.9), and its ceiling effect was 32%.

**Table 4:** Distribution of Health-Related Quality of Life (HRQL) scores

Distribution of scores	EQ-5D-Y Utility Index	EQ-VAS^a^	PROMIS-PAIS^b^ Raw score
Sample	119	119	75
Theoretical Range	+ 1, − 0.5392	100, 0	8, 40
	Best–worst	Best–worst	Best–worst
Observed Range	+ 1, + 0.5095	100, 25	8, 29
Floor effect	0.0%	0.0%	0.0%
Ceiling effect	60.3%	23.5%	32.0%
Mean (SD)	0.93 (0.11)	84.3 (17.1)	11.9 (4.9)
Missing	3 (2.5%)	0 (0.0%)	0 (0.0%)

^a^EQ-VAS: EuroQol-Visual Analogue Scale

^b^PROMIS-PAIS: Patient-Reported Outcomes Measurement Information System-Pediatric Asthma Impact Scale

Table 5 presents the multitrait-multimethod matrix between EQ-5D-Y, PROMIS-PAIS, and ACQ. For the two correlations previously hypothesized as moderate (convergent validity) we obtained a coefficient of 0.38 between the PROMIS-PAIS’ raw score and the EQ-5D-Y utility index, and of 0.35 between the EQ-5D-Y dimension “problems doing usual activities” and the ACQ item “how limited were you in your activities because of your asthma?”. Regarding discriminant validity, the two relationships hypothesized as weak obtained a correlation of 0.28 between the ACQ and the EQ-5D-Y utility index, and 0.17 between the EQ-5D-Y dimension “problems doing usual activities” and the PROMIS-PAIS item “it was hard for me to play sports or exercise because of my asthma”. The correlation of the EQ-VAS with the other two asthma-specific instruments was lower than that obtained with the EQ-5D-Y utility index.

**Table 5:** Multitrait-multimethod matrix between the EQ-5D-Y, the Pediatric Asthma Impact Scale and the Asthma Control Questionnaire

	EQ-5D-Y Utility index	EQ-VAS^c^ Visual Analogue Scale	EQ-5D-Y Dimension (Problems doing usual activities)	ACQ^d^ Global Score	ACQ Item (Limited in activities because of asthma)
PROMIS-PAIS Global Score	0.38^a^ n = 74 (p = .001) CI [0.56 to 0.16]	0.16 n = 75 (p = .158) CI [0.38 to 0.06]	0.33 n = 75 (p = .003) CI [0.12 to 0.52]	0.37 n = 73 (p = .001) CI [0.15 to 0.55]	0.29 n = 73 (p = .014) CI [0.06 to 0.48]
PROMIS-PAIS^e^ Item (Hard to play sports or exercise because of asthma)	0.25 n = 74 (p = .033) CI [0.45 to 0.02]	0.16 n = 75 (p = .158) CI [0.38 to 0.06]	0.17^b^ n = 75 (p = .151) CI [ 0.06 to 0.38]	0.31 n = 73 (p = .008) CI [0.08 to 0.50]	0.30 n = 73 (p = .011) CI [0.07 to 0.49]
ACQ Global Score	0.28^b^ n = 113 (p = .002) CI [0.44 to 0.10]	0.26 n = 116 (p = .004) CI [0.43 to 0.09]	0.30 n = 115 (p = .001) CI [0.12 to 0.46]	1	0.79 n = 116 (p = .000) CI [0.71 to 0.85]
ACQ Item (Limited in activities because of asthma)	0.36 n = 113 (p = .000) CI [0.51 to 0.18]	0.26 n = 116 (p = .005) CI [0.42 to 0.08]	0.35^a^ n = 115 (p = .000) CI [0.18 to 0.50]	0.79 n = 116 (p = .000) CI [0.71 to 0.85]	1

Correlation coefficients are presented without a sign, since it only reflects that instruments’ scores are in the same or in the opposite direction

^a^Correlation hypothesized as moderate (0.31–0.60)

^b^Correlation hypothesized as weak (≤ 0.30)

^**c**^EQ-VAS: EuroQol-Visual Analogue Scale

^d^ACQ: Asthma Control Questionnaire

^e^PROMIS-PAIS: Patient-Reported Outcomes Measurement Information System-Pediatric Asthma Impact Scale

Table 6 shows statistically significant differences in some dimensions of the EQ-5D-Y, the utility index, and the EQ-VAS, among known groups defined by their asthma control with the ACQ, frequency of SABA use in the last 4 weeks, and second-hand smoke exposure. The effect size of almost all these differences in both the EQ-5D-Y utility index and the EQ-VAS was ≥ 0.5, indicating moderate magnitude. The largest magnitude of the difference with the PROMIS-PAIS raw score was 1.11 among asthma control groups (p = 0.02).

**Table 6:** Comparison of Health-Related Quality of Life (HRQL) between known groups measured at baseline

	% of participants reporting problems in each dimension (n of participants with problems/n of participants without problems)					EQ-5D-Y Utility Index	EQ-VAS^a^	PROMIS PAIS^b^ raw Score
	Mobility	Looking after myself	Doing usual activities	Having pain/discomfort	Feeling worried/sad/unhappy	Mean (SD) n = 116	Mean (SD) n = 119	Mean (SD) n = 75
*Asthma control—ACQ*^c^								
Well controlled	4.4% (3/65)	1.5% (1/67)	20.6% (14/54)	14.7% (10/58)	7.4% (5/63)	.94 (.12)	88.2 (14.4)	10.9 (4.4)
Intermediate	0.0% (0/23)	4.3% (1/22)	39.1% (9/14)	21.7% (5/18)	8.7% (2/21)	.91 (.11)	78.6 (20.6)	11.3 (3.3)
Not well controlled	13.0% (3/20)	4.3% (1/22)	45.8% (11/13)	24.0% (6/19)	8.0% (2/23)	.89 (.12)	77.9 (18.5)	16.2 (6.2)
*P* value	.12	.64	.04	.52	.98	.06	< .006	.002
ES^d^ [95% CI^e^]	N/A^f^	N/A^f^	N/A^f^	N/A^f^	N/A^f^	− 0.38 [− 0.87 to 0.1]	0.66 [0.19 to 1.13]	1.11 [0.45 to 1.76]
*Asthmatic exacerbations (last 6 months)*								
No	6.3% (4/60)	1.5% (1/64)	23.1% (15/50)	13.6% (9/57)	6.1% (4/62)	0.94 (0.11)	84.6 (18.3)	11.3 (4.8)
Yes	3.8% (2/51)	3.8% (2/50)	35.8% (19/34)	22.6% (12/41)	9.4% (5/48)	0.91 (0.12)	84.0 (15.6)	12.9 (5.0)
*P* value	.55	.43	.13	.20	.49	.12	.46	.15
ES [95% CI]	N/A^f^	N/A^f^	N/A^f^	N/A^f^	N/A^f^	− 0.24 [− 0.61 to 0.13]	0.03 [− 0.33 to 0.4]	0.35 [− 0.13 to 0.82]
*Frequency of SABA*^g^* use reported by patients (last 4 weeks)*								
Less than once per week	4.3% (3/66)	1.4% (1/69)	18.6% (13/57)	11.3% (8/63)	5.6% (4/67)	0.95 (0.09)	88.4 (14.2)	11.4 (4.9)
Once or more per week	6.7% (3/42)	4.5% (2/42)	46.7% (21/24)	28.9% (13/32)	11.1% (5/40)	0.88 (0.14)	77.2 (19.4)	12.7 (5.0)
*P* value	.59	.31	.001	.02	.28	.004	.001	.27
ES [95% CI]	N/A^f^	N/A^f^	N/A^f^	N/A^f^	N/A^f^	− 0.59 [− 0.98 to − 0.2]	0.68 [0.30 to 1.07]	0.26 [− 0.21 to 0.73]
*Second-hand smoke exposure*								
Not exposed	5.1% (4/75)	1.3% (1/79)	27.5% (22/58)	17.3% (14/67)	9.9% (8/73)	0.92 (0.11)	85.6 (15.4)	11.6 (4.7)
Exposed	12.5% (2/14)	13.3% (2/13)	43.8% (7/9)	37.5% (6/10)	6.3% (1/15)	0.87 (0.16)	73.5 (19.7)	13.7 (6.6)
*P* value	.27	.01	.20	.07	.65	.35	.02	.21
ES [95% CI]	N/A^f^	N/A^f^	N/A^f^	N/A^f^	N/A^f^	− 0.45 [− 1.01 to 0.1]	0.75 [0.21 to 1.30]	0.42 [− 0.23 to 1.07]

^a^EQ-VAS: EuroQol-Visual Analogue Scale

^b^PROMIS-PAIS: Patient-Reported Outcomes Measurement Information System-Pediatric Asthma Impact Scale

^c^ACQ: Asthma Control Questionnaire

^d^ES: effect size

^e^CI: interval confidence

^f^N/A: not applicable

^g^SABA: Short-Acting β-Agonists

Table 7 shows test–retest reproducibility results for the subsamples of stable patients and responsiveness results for the subsamples of patients with worsening and improvement, defined according to the changes on the EQ-VAS or the ACQ. Of the 62 participants who answered the EQ-5D-Y twice, after excluding three for missing values, 59 were finally distributed into the subsamples (24 stable, 17 worsened and 18 improved). Only 46 participants answered both the PROMIS-PAIS and the EQ-5D-Y twice. The subsamples defined according to the ACQ were smaller, since we excluded nine patients whose telephone interview was more than 90 days apart from their app response. For the stable EQ-VAS subsample, the mean change was of 0.01 in the EQ-5D-Y utility index and -0.5 in the PROMIS-PAIS raw score with ICCs of 0.81 and 0.89 respectively, indicating high agreement between both evaluations (reproducibility). Regarding responsiveness, though change of means was not statistically significant, the effect size was of moderate and large magnitude for the EQ-5D-Y utility index and PROMIS-PAIS raw score (0.68 and 1.08 respectively) among patients in the worsening or improvement subsamples, analyzed together.

**Table 7:** Evaluation of reproducibility and responsiveness of EQ-5D-Y’s utility index and PROMIS-PAIS

	Stable subsample	Worsening subsample	Improvement subsample
*Subsamples defined according to the EQ-VAS*^a^			
EQ-5D-Y utility Index			
n	24	17	18
1st administration, mean (SD)	0.94 (0.11)	0.98 (0.04)	0.90 (0.12)
2nd administration, mean (SD)	0.95 (0.09)	0.95 (0.10)	0.94 (0.10)
Change, mean (SD)	0.01 (0.08)	− 0.04 (0.09)	0.04 (0.11)
*P* value	.50	.12	.18
Effect size	0.14	0.68^d^	
ICC^b^	0.81	N/A^c^	N/A^c^
PROMIS-PAIS raw score^e^			
n	19	12	15
1st administration, mean (SD)	39.0 (7.4)	41.4 (7.8)	38.0 (6.6)
2nd administration, mean (SD)	39.5 (9.8)	36.5 (5.8)	37.6 (6.0)
Change, mean (SD)	− 0.5 (5.5)	5.0 (8.7)	0.4 (10.1)
*P* value	.71	.07	.88
Effect size	0.09	1.08^d^	
ICC^b^	0.89	N/A^c^	N/A^c^
*Subsamples defined according to the ACQ*^f^			
EQ-5D-Y utility Index			
n	29	6	16
1st administration, mean (SD)	0.94 (0.10)	0.92 (0.15)	0.95 (0.08)
2nd administration, mean (SD)	0.96 (0.09)	0.88 (0.14)	0.95 (0.08)
Change, mean (SD)	0.02 (0.08)	− 0.04 (0.13)	0.00 (0.11)
*P* value	.31	.50	.94
Effect size	0.19	0.78^d^	
ICC^b^	0.79	N/A^c^	N/A^c^
EQ-VAS^a^			
n	30	6	16
1st administration, mean (SD)	88.9 (14.1)	78.5 (22.3)	80.4 (16.3)
2nd administration, mean (SD)	90.8 (11.7)	83.0 (19.3)	83.1 (18.1)
Change, mean (SD)	2.0 (12.5)	4.5 (24.0)	2.7 (20.2)
*P* value	.40	.67	.59
Effect size	0.16	1.15^d^	
ICC^b^	0.70	N/A^c^	N/A^c^
PROMIS-PAIS raw score^e^			
n	25	6	11
1st administration, mean (SD)	36.5 (4.0)	48.0 (10.1)	42.1 (9.0)
2nd administration, mean (SD)	36.7 (5.2)	46.6 (11.8)	38.5 (7.1)
Change, mean (SD)	− 0.2 (4.6)	1.4 (13.8)	3.7 (10.3)
*P* value	.83	.82	.27
Effect size	0.04	1.28^d^	
ICC^b^	0.68	N/A^c^	N/A^c^

^a^EQ-VAS: EuroQol-Visual Analogue Scale

^b^ICC: Intra-class Correlation Coefficient

^c^N/A: not applicable

^d^Effect size of change calculated for the worsening and improvement subsamples together

^e^PROMIS-PAIS: Patient-Reported Outcomes Measurement Information System-Pediatric Asthma Impact Scale

^f^ACQ: Asthma Control Questionnaire

The agreement (reproducibility) in the stable subsample defined according to ACQ for the EQ-5D-Y utility index, EQ-VAS and the PROMIS-PAIS raw score measured with the ICC was 0.79, 0.70 and 0.68, respectively; and regarding responsiveness, the effect size of change was large (0.78, 1.15 and 1.28) among patients from the worsening or improvement subsamples, analyzed together.

## Discussion

We found the EQ-5D-Y to be feasible and easy to administer via a smartphone application, but with a high ceiling effect (60.3% of participants reported no problem in any dimension). This generic preference-based instrument showed good validity, considering the moderate correlation between the EQ-5D-Y utility index and the PROMIS-PAIS raw score, and the discrimination among known groups based on the ACQ, frequency of Short-Acting Beta-Agonists (SABAs), and second-hand smoke exposure. Test–retest reproducibility among the stable subsamples indicated high reliability; and the magnitude of change observed between the first and second evaluation in the worsening or improvement subsamples may suggest its responsiveness, but the differences were not statistically significant.

Feasibility of the EQ-5D-Y was indicated by its high response rate at baseline (97.5%): of the 119 participants who downloaded the app, 116 responded the EQ-5D-Y entirely. These results are similar to the 96% response rate reported in the abovementioned cross-sectional Swedish study on children and adolescents with asthma [42]. The flexibility of the administration through the app benefits our completion results, especially for the EQ-VAS with a completion rate of almost 100%, compared with 91% and 86.2% reported in studies where the EQ-5D-Y was administered in paper format to children with chronic conditions [43] and schoolchildren [44], respectively. The losses to follow-up (44 participants who downloaded the app but did not follow until month 6) are likely related to major misunderstandings regarding the app, such us patients thinking that it only had to be answered once and then deleted [52]. On the other hand, 8 patients who continued using the app did not answer the EQ-5D-Y at month 6, which is a response rate of 88.5%. This high level of completion in both administrations could be explained by its low response burden: response times have been estimated on 1.25 min for EQ-5D-Y web version [34]. These results support the feasibility of the EQ-5D-Y when it is administered through a smartphone application.

Regarding the distribution of EQ-5D-Y results, the ceiling effect in the sample was high for the utility index (60.3%) and for three out of its five dimensions, which exceeded 90% of participants reporting no problem (“mobility”, “looking after myself”, and “feeling worried/sad/unhappy”). This high ceiling effect in our sample could be partly explained by the considerable proportion of participants with well-controlled symptoms of asthma (58.6%). Furthermore, a similar ceiling effect, above 80%, has been also reported for the “mobility” and “looking after myself” dimensions in other studies with children or adolescents with asthma [42] and other chronic conditions [43, 44]. These ceiling effects are very high considering the established recommendation of 15% for HRQL scores [70]. However, this is a general standard, as there are none specifically for children. A higher ceiling effect could be expected in children, taking into account their capacity of adaptation to chronic diseases and treatment routines, known as the well-being paradox or response shift effect [71, 72]. The prevalence of problems reported in the dimensions of “doing usual activities” (29.6%) and “having pain or discomfort” (18.1%) are consistent with previous studies [42, 44, 73] where the EQ-5D-Y has been used to describe the impact of asthma on children and adolescents. Studies using the Pediatric Asthma Quality of Life Questionnaire (PAQLQ) [42, 74] or the Child Health Questionnaire (CHQ-CF87) [8] also highlighted the physical activity limitation, while another study measuring HRQL with the PedsQL™ [10] concludes that asthma has an impact on physical, emotional, and school performance. In our study, participants reported a low percentage of problems in the dimension of “Feeling worried, sad or unhappy” (7.6%).

Construct validity of the EQ-5D-Y was evaluated by exploring its relationships as a generic measure with the asthma-specific PROMIS-PAIS, as there is no gold standard for HRQL assessment in pediatric asthma. The correlation between the EQ-5D-Y utility index and the PROMIS-PAIS raw score is moderate in our study, showing that both instruments are capturing the HRQL impact of asthma although not similarly, mainly due to differences between generic and asthma-specific approaches, and to a lesser extent due to differences between the psychometric and econometric development. In our study, these questionnaires were administered at different periods (EQ-5D-Y at baseline and PROMIS-PAIS at month 4), which may have produced an underestimation of their correlations. Previous studies have remarked that asthma-specific HRQL instruments measure similar contents to those covered by asthma control questionnaires [75, 76], with a high correlation between them (0.78) [76], proposing generic HRQL instruments to add broader domains which are also important to patients with asthma [77]. However, in our study the correlation of the ACQ with the PROMIS-PAIS was moderate (0.37, 95% CI 0.15–0.55), and not significantly stronger than that of the ACQ with the EQ-5D-Y index (0.28, 95% CI 0.44–0.10).

As we hypothesized, the correlation of the EQ-5D-Y dimension “problems doing usual activities” with the ACQ item “how limited were you in your activities because of your asthma?” was moderate, and with the PROMIS-PAIS item “it was hard for me to play sports or exercise because of my asthma” was insignificant. This was similar to the correlation of 0.21 between “mobility” (EQ-5D-Y) and “physical wellbeing” (KIDSCREEN) reported in children with diabetes [39], arguing that some KIDSCREEN items consider high energy activities such as “have you been physically active (e. g. running, climbing, biking)?”.

Furthermore, the results on discrimination capacity of the EQ-5D-Y utility index and EQ-VAS among most of the selected known groups confirm the hypothesized direction. Although these groups are well known in adults, as far as we know there are no EQ-5D-Y studies evaluating them in children: the EQ-5D was able to detect differences between groups defined by the ACQ [65], and presented a significant association with second-hand smoke exposure [67]. These findings provide evidence of the EQ-5D-Y’s ability to detect differences in these known groups, indicating a good construct validity of the instrument, which presented a similar discriminant capacity to the PROMIS-PAIS among groups except for those defined by the ACQ. The PROMIS-PAIS raw score showed, as expected, greater differences than EQ-5D-Y between patients with well- and not well-controlled asthma defined by ACQ (large effect size of 1.11).

Regarding content validity, it is important to mention that the EQ-5D-Y does not cover key aspects of children’s HRQL such as social, emotional, or school impact, unlike other generic HRQL instruments that do include them. For example, the KIDSCREEN [78] considers “autonomy and relationships with parents”, “social support”, “relationship with friends”, and “school environment”; the Child Health Questionnaire [79] includes “role/social emotional and behavioral functioning”; and the Child Health Utility 9D [80] asks about “school, daily routine and activities”.

Our study shows good reproducibility of the EQ-5D-Y utility index and EQ-VAS according to the established standard [70] (ICC equal or greater than 0.70), which was consistent with the ICC of the EQ-5D-Y unweighted summary score (0.83) reported in a study with children with type 1 diabetes mellitus for the EQ-5D-Y summary score [39]. Two studies reported from poor to substantial agreement according to the EQ-5D-Y dimension, with Kappa coefficients ranging from 0.003 to 0.67 [33] and from 0.199 to 0.653 [41]. The period of time between test and retest in these two studies was short (7–10 days and 48 h). In our case, a 6-month time interval between evaluations could underestimate the reproducibility, but this effect could be compensated by the selection of stable patients. Reproducibility of the PROMIS-PAIS raw score was high in the stable subsample defined with the EQ-VAS (ICC of 0.89) and acceptable in the subsample defined with the ACQ (ICC of 0.68). We did not find previous studies evaluating reproducibility of the PROMIS-PAIS.

Regarding responsiveness of the EQ-5D-Y utility index, we obtained a moderate to large capacity of the instrument to detect change over time. As expected, a larger capacity of detecting change was observed with the asthma-specific instrument PROMIS-PAIS. The EQ-5D-Y unweighted summary score has demonstrated its ability to detect improvement of a moderate magnitude in children and adolescents with type 1 diabetes mellitus (n = 58) [39]. A study of children with idiopathic scoliosis (n = 110) [40] demonstrated the responsiveness of the EQ-5D-Y index constructed with adult value sets, obtaining a large worsening and a small improvement in subsamples defined according to the global rating of health scale.

### Strengths and limitations

The Asthma Research in Children and Adolescents (ARCA) study provides a complete database with repeated administration of HRQL instruments and disease-related variables that allow the assessment of EQ-5D-Y’s (self-response and proxy version) psychometric properties. The administration of the PROMIS-PAIS allowed us to assess the construct validity of the generic EQ-5D-Y, comparing it with an asthma-specific HRQL instrument. To the best of our knowledge, this is the first study evaluating the psychometric properties of the EQ-5D-Y using the value sets for children, and also the first one including the proxy version to collect HRQL of children with asthma younger than 8 years old.

The effect of the SARS-CoV-2 pandemic on this study deserves a comment. Of the 119 patients included in this study, there were only 8 patients recruited after the SARS-CoV-2 lockdown (between March 2020 and the closing of the data base in June 2020). Of the patients recruited before lockdown, 19 should have responded the 6-month follow-up during this 4-month period, but only 6 answered the EQ-5D-Y. Considering the low number of participants who had to answer during the SARS-CoV-2 lockdown, results are not likely to be affected by the pandemic. On the other hand, 18 participants reported during the telephone interviews having suffered SARS-CoV-2, but only two had been diagnosed before closing the data base of this analysis.

Some limitations should be mentioned. First, this is a secondary objective of the ARCA study, primarily designed for purposes other than the evaluation of the psychometric properties of EQ-5D-Y. Second, the small sample size in the proxy response (n = 38) prevented an independent, complete analysis for self-response and proxy-response versions. However, the examination of the distribution of each version showed a similar pattern (see Tables S1 and S2 in the Additional file 1). Third, the skewness (high ceiling effect) of the EQ-5D-Y utility index could have affected the construct validity results and also hinder the detection of improvement. The EuroQol research foundation’s younger populations working group recently developed the new version with 5 levels (EQ-5D-Y-5L) [81], since expanding the number of severity levels might reduce ceiling effects and improve sensitivity. Fourth, responsiveness results should be interpreted with caution considering the low number of participants evaluated due to the classification into two subsamples (stable and changed), and also in part the losses to follow-up (n = 44). The latter could have introduced an attrition bias; in fact, differences found in the asthmatic exacerbations in the last 6 months reported at baseline suggest that the participants could be healthier than those who dropped out (see Table S3 in the Additional file 1). Fifth, because the ARCA project only included patients with mild-to-moderate persistent asthma, the generalizability of our results to those with intermittent or severe persistent asthma is uncertain. Its generalizability is also uncertain to other chronic conditions.

## Conclusion

Despite the limitations discussed above, our results provide considerable evidence supporting the appropriate psychometric properties of the EQ-5D-Y in children with persistent asthma. In conclusion, these findings suggest that the EQ-5D-Y is a feasible, valid, and reliable instrument for evaluating HRQL in children and adolescents with mild-to-moderate persistent asthma when it is self-responded by 8–12 years old patients and through proxy response by parents of children under 8 years old. However, the EQ-5D-Y’s high ceiling effect found in our sample suggests that it may be more suitable for patients with severe asthma and a higher presence of problems. Further head-to-head comparisons of the three-level and the new five-level version of the EQ-5D-Y in children with asthma are needed to examine to what extent expanding the number of response levels decreases ceiling effect and increases responsiveness. The recent development of the value sets for Spanish children [50] following the international protocol [48] will allow calculating quality-adjusted life-years (combining both the quantity and quality of life) for economic evaluations, since it is a preference-based health status measure. It is a promising instrument to compare the efficiency of different programs or treatment strategies, helping prioritization and investment at different levels. Given its short and easy administration, the EQ-5D-Y is a practical instrument to be used for monitoring patients through the use of smartphone applications.

## Supplementary Information

**Additional file 1: Table S1.** Distribution of Health-Related Quality of Life (HRQL) scores, self-response version (n=81).** Table S2**. Distribution of Health-Related Quality of Life (HRQL) scores, proxy-response version (n = 38).** Table S3**. Demographic characteristics of participants who completed the 6-month follow-up evaluation and those who did not.

## Data Availability

The datasets used and/or analysed during the current study are available from the corresponding author upon reasonable request.

## Abbreviations

ARCA: Asthma Research in Children and Adolescents
HRQL: Health-Related Quality of Life
ACQ: Asthma Control Questionnaire
CHQ-CF87: Child Health Questionnaire
CHU-9D: Child Health Utility 9D
EQ-VAS: EuroQol-Visual Analogue Scale
HUI: Health Utility Index
SMS: Short Message Service
ICC: Intra-class Correlation Coefficient
PedQL: Pediatric Quality of Life Inventory
PROMIS PAIS: Patient-Reported Outcomes Measurement Information System-Pediatric Asthma Impact Scale
PROMS: Patient-Reported Outcome Measures
SABA: Short-Acting β-Agonists
SD: Standard deviations
PAQLQ: Pediatric Asthma Quality of Life Questionnaire
