# Supplementary material for: Measurement properties of the EQ-5D-Y administered through a smartphone app in children with asthma: a longitudinal questionnaire study
Source: Health Qual Life Outcomes. 2022 Mar 28;20:51. doi: 10.1186/s12955-022-01955-5 (PMC8959271; doi:10.1186/s12955-022-01955-5)
Supplement: Supplementary file 1 — Additional file 1: Table S1. Distribution of Health-Related Quality of Life (HRQL) scores, self-response version (n=81). Table S2. Distribution of Health-Related Quality of Life (HRQL) scores, proxy-response version (n = 38). Table S3. Demographic characteristics of participants who completed the 6-month follow-up evaluation and those who did not. [file 12955_2022_1955_MOESM1_ESM.docx]

# Table S1. Distribution of Health-Related Quality of Life (HRQL) scores, self-response version (n=81)

| **Distribution of scores** | **EQ-5D-Y I Utility Index** | **EQ-VAS^a^** | **PROMIS-PAIS^b^ Raw score** |
| --- | --- | --- | --- |
| Sample | 81 | 81 | 56 |
| Theoretical Range | +1, -0.5392 | 100, 0 | 8, 40 |
|  | Best-worst | Best-worst | Best-worst |
| Observed Range | +1.0, +0.51 | 100, 25 | 8, 29 |
| Floor effect | 0.0% | 0.0% | 0.0% |
| Ceiling effect | 55.0% | 24.7% | 28.6% |
| Mean (SD) | 0.92 (0.12) | 81.8 (18.8) | 11.7 (4.7) |
| Missing | 1 (1.2%) | 0 (0.0%) | 0 (0.0%) |

^a^EQ-VAS: EuroQol-Visual Analogue Scale

^b^PROMIS-PAIS: Patient-Reported Outcomes Measurement Information System - Pediatric Asthma Impact Scale

# Table S2. Distribution of Health-Related Quality of Life (HRQL) scores, proxy-response version (n= 38)

| **Distribution of scores** | **EQ-5D-Y**  **Utility Index** | **EQ-VAS^a^** | **PROMIS-PAIS^b^ Raw score** |
| --- | --- | --- | --- |
| Sample | 38 | 38 | 19 |
| Theoretical Range | +1, -0.5392 | 100, 0 | 8, 40 |
|  | Best-worst | Best-worst | Best-worst |
| Observed Range | +1.0, +0.57 | 100, 50 | 8, 24 |
| Floor effect | 0.0% | 0.0% | 0.0% |
| Ceiling effect | 72.2% | 21.1% | 42.1% |
| Mean (SD) | 0.95 (0.11) | 89.7 (11.1) | 12.4 (5.6) |
| Missing | 2 (5.3%) | 0 (0.0%) | 0 (0.0%) |

^a^EQ-VAS: EuroQol-Visual Analogue Scale

^b^PROMIS-PAIS: Patient-Reported Outcomes Measurement Information System - Pediatric Asthma Impact Scale

**Table S3.** Demographic characteristics of participants who completed the 6-month follow-up evaluation and those who did not.

|  | **Baseline and**  **6-month follow-up**  **(n = 62)** | **Only baseline**  **(n = 52)** | ***P* value** |
| --- | --- | --- | --- |
| **Age**, mean (SD) | 9.1 (1.5) | 8.8 (1.9) | .37 |
| 6 – 7 | 16 (25.8%) | 22 (42.3%) | .06 |
| 8 – 11 | 46 (74.2%) | 30 (57.7%) |  |
| **Sex,** n (%) |  |  |  |
| Girls | 23 (37.1%) | 22 (42.3%) | .57 |
| Boys | 39 (62.9%) | 30 (57.7%) |  |
| **Symptoms Control**  **ACQ^a^**, mean (SD) | 0.80 (1.00) | 0.90 (0.91) | .43 |
| Well controlled (< 0.75) | 37 (61.7%) | 27 (52.9%) | .39 |
| Intermediate (0.75 – 1.5) | 13 (21.7%) | 10 (19.6%) |  |
| Not well controlled (> 1.5) | 10 (16.7%) | 14 (27.5%) |  |
| **Asthmatic exacerbations (last 6 months)** |  |  |  |
| Yes | 21 (33.9%) | 32 (61.5%) | .003 |
| No | 41 (66.1%) | 20 (38.5%) |  |
| **Number of prescribed SABA^b^** |  |  |  |
| 0 | 5 (8.3%) | 4 (7.8%) | .90 |
| 1 | 53 (88.3%) | 46 (90.2%) |  |
| 2 | 2 (3.3%) | 1 (2.0%) |  |
| **Frequency of SABA inhaler use (previous 4 weeks)** |  |  |  |
| No use | 5 (8.3%) | 4 (7.8%) | .63 |
| Less than once per week | 35 (58.3%) | 24 (47.1%) |  |
| Once or twice per week | 13 (21.7%) | 14 (27.5%) |  |
| Almost every day / Every day | 7 (11.1%) | 9 (17.6%) |  |
| **Secondhand smoke exposure** |  |  |  |
| Not exposed | 46 (90.2%) | 32 (78.0%) | .11 |
| Exposed (home, car or both) | 5 (9.8%) | 9 (22.0%) |  |
| Missing | 11 | 11 |  |

^a^ACQ: Asthma Control Questionnaire

^b^SABA: Short-Acting β-Agonists
